# Complete organelle genomes of the threatened aquatic species *Scheuchzeria palustris* (Scheuchzeriaceae): Insights into adaptation and phylogenomic placement

**DOI:** 10.1002/ece3.70248

**Published:** 2024-08-31

**Authors:** Xiang‐Yan He, Jin‐Ming Chen, Zhi‐Zhong Li

**Affiliations:** ^1^ Collaborative Innovation Center of Recovery and Reconstruction of Degraded Ecosystem in WanjiangBasin Co‐Funded by Anhui Province and Ministry of Education of the People's Republic of China, School of Ecology and Environment Anhui Normal University Wuhu China; ^2^ Aquatic Plant Research Center, Wuhan Botanical Garden Chinese Academy of Sciences Wuhan China; ^3^ University of Chinese Academy of Sciences Beijing China

**Keywords:** mitogenome, positive selection, RNA editing, substitution rate

## Abstract

*Scheuchzeria palustris*, the only species in the Scheuchzeriaceae family, plays a crucial role in methane production and transportation, influencing the global carbon cycle and maintaining ecosystem stability. However, it is now threatened by human activities and global warming. In this study, we generated new organelle genomes for *S. palustris*, with the plastome (pt) measuring 158,573 bp and the mitogenome (mt) measuring 420,724 bp. We predicted 296 RNA editing sites in mt protein‐coding genes (PCGs) and 142 in pt‐PCGs. Notably, abundant RNA editing sites in pt‐PCGs likely originated from horizontal gene transfer between the plastome and mitogenome. Additionally, we identified positive selection signals in four mt‐PCGs (*atp4*, *ccmB*, *nad3*, and *sdh4*) and one pt‐PCG (*rps7*), which may contribute to the adaptation of *S. palustris* to low‐temperature and high‐altitude environments. Furthermore, we identified 35 mitochondrial plastid DNA (MTPT) segments totaling 58,479 bp, attributed to dispersed repeats near most MTPT. Phylogenetic trees reconstructed from mt‐ and pt‐PCGs showed topologies consistent with the APG IV system. However, the conflicting position of *S. palustris* can be explained by significant differences in the substitution rates of its mt‐ and pt‐PCGs (*p* < .001). In conclusion, our study provides vital genomic resources to support future conservation efforts and explores the adaptation mechanisms of *S. palustris*.

## Graphical abstract

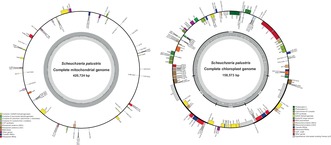

## 1 INTRODUCTION

*Scheuchzeria palustris*, the sole species of the monotypic family Scheuchzeriaceae, exhibits a circumpolar distribution, which predominantly distributes in the temperate and frigid marshy habitats of the Northern Hemisphere, encompassing the boreal and temperate zones of Finland, Canada, the United States, and China (Tallis & Birks, 1965). As a typical plant of marshy swamp wetlands, it significantly influences methane production and transportation, thereby playing a pivotal role in regulating the global carbon cycle and maintaining ecosystem stability (Dorodnikov et al., 2011). Owing to excessive human activities and global warming, the habitat of *S. palustris* has been severely degraded, leading to a sharp decline in its wild populations and it has been classified as a threatened species in many European countries (Smith et al., 2021). In China, *S. palustris* is sporadically found in the alpine or sub‐alpine swamps of the Changbai Mountain region, near the southernmost boundary of its range and extremely vulnerable to climate warming, which leads to its inclusion as a Grade II protected plant in China (Lu et al., 2021). Previous studies on *S. palustris* have primarily focused on its ecology (Moore, 1955), floral development (Volkova et al., 2016), and phylogeny (Li et al., 2023; Ross et al., 2016). However, there is still a lack of basic genetic resources for this unique aquatic plant, which limits further research and conservation efforts for this threatened species.

Chloroplasts (pt) and mitochondria (mt) are considered semi‐autonomous organelles with independent genetic material in eukaryotic cells (Birky, 2001). This unique feature makes them invaluable in various applications, including phylogeny (Li et al., 2020; Mwanzia et al., 2020), inheritance patterns (Birky, 2001; Park et al., 2021), and adaptive evolution (Yang, Fu, et al., 2022; Yang, Zhao, et al., 2022). These organelles play key roles in cellular processes in plants, particularly in energy metabolism. Chloroplasts, the primary sites of photosynthesis in plant cells, convert solar energy into carbohydrates and oxygen, while mitochondria serve as the cell's energy center, supplying the energy required for numerous cellular processes in plants (Lai et al., 2022; Liberatore et al., 2016; Tang et al., 2020). The number of documented plastomes significantly surpasses that of mitogenomes due to the relatively simpler structure (ca.120–130 genes) and smaller size (ca.120–170 kb) of plastomes in angiosperms, which typically exhibit a conserved four‐part architecture containing inverted repeats (IRs) and single‐copy regions (Henry, 2005; Jansen & Ruhlman, 2012). In contrast, plant mitogenomes are generally larger and more complex, characterized by varying genome sizes (ca. 100–10,000 kb), diverse conformations, low gene densities, and an abundance of repetitive sequences and RNA editing events (Jansen & Ruhlman, 2012), making the conformation of plant mitogenome challenging (Gualberto et al., 2014). Moreover, horizontal gene transfer (HGT) of DNA from plastome to mitogenome in angiosperms is a common phenomenon, which might affect organelle gene function and expression (Kim et al., 2023; Straub et al., 2013), thereby influencing adaptation to stress resistance (i.e., cold and high altitude).

Due to the ease of assembling plastomes, nearly 10,000 plant plastomes have been reported and widely applied to resolve phylogeny at different taxonomic levels (Wang, Kan, et al., 2024; Wang, Liu, et al., 2024). Our previous research reported the plastome of *S. palustris* and explored the dynamic history of plastome structure across Alismatidae (Li et al., 2023). However, research on the mitogenome of *S. palustris* remains limited. With the recent advancements in sequencing technologies and assembly methods, obtaining plant mitogenomes has become feasible (Bi, Shen, et al., 2024; Bi, Sun, et al., 2024; He et al., 2024), which has provided us with the opportunity to investigate mitogenomic plant phylogeny and cytoplasmic evolution through the growing number of reported plant mitogenomes. In this study, we newly assembled two organelle genomes of *S. palustris* using High‐Fidelity (HiFi) sequencing. Our objectives were to: (1) analyze the repeated elements and RNA‐editing sites; (2) identify HGT events between plastome and mitogenome; (3) infer the phylogenetic relationships based on the two different organelle genomes and (4) explore heterogeneity associated with maternally inherited organelle. Our results will contribute to understanding the organelle genome evolution in the threatened aquatic plant *S. palustris* and provide vital genomic resources and scientific support for its future conservation.

## 2 MATERIALS AND METHODS

### 2.1 Plant materials and sequencing

The cultivated sample of *S. palustris* was collected from Wuhan Botanical Garden, the Chinese Academy of Sciences in China. Total genomic DNA was isolated from young leaves using the modified CTAB method (Doyle & Doyle, 1987) for HiFi sequencing. The construction of the sequencing library followed the standard protocol of Pacific Biosciences, and HiFi sequencing was conducted on the PacBio Sequel2 platform. In total, ca. 3 Gb of HiFi reads (genome size 0.54G; https://cvalues.science.kew.org/) were generated and applied for subsequent analyses.

### 2.2 Organelle genome assembly and annotation

To obtain the complete mitogenome and plastome of *S. palustris*, all HiFi reads were de novo assembled using PMAT v1.5.1 (Bi, Shen, et al., 2024; Bi, Sun, et al., 2024) with the autoMito module and default parameters. The initial assembly graph of the organelle genomes was then manually edited with Bandage v0.8.1 (Wick et al., 2015). This process resulted in the generation and identification of two circular contigs: contig‐mt (420,724 bp) and contig‐pt (158,573 bp). These contigs were confirmed by BLAST analysis using conserved mitochondrial and plastid protein‐coding genes (PCGs) from *Zostera marina* (NC_035345.1/NC_036014.1) and *Butomus umbellatus* (NC_021399.1/NC_051949.1). Additionally, the online tool GeSeq (Tillich et al., 2017) was used for the initial annotation of the mitogenome and plastome of *S. palustris*, with *Z. marina* and *B. umbellatus* serving as references. Annotations were further manually verified and corrected using Geneious v5.6.4 (Biomatters Ltd., Auckland, New Zealand). The annotated mitogenome and plastome have been deposited into GeneBank (Table S1) and a graphic representation of the circular organelle genome map was visualized by OGDRAW (Greiner et al., 2019).

### 2.3 Prediction of RNA editing sites and codon usage

To identify RNA‐editing sites in two organelle genomes PCGs of *S. palustris*, RNA‐seq data were retrieved from GenBank with the accession Nos. SRR18139151 and SRR21619434. After filtering out poor‐quality reads using Fastp v0.20.0 (Chen et al., 2018) with default settings, the high‐quality reads were remapped to pt‐PCGs and mt‐PCGs of *S. palustris* using BWA v.0.7.17‐r1188 (Li, 2013), respectively. To enhance the coverage and depth for each locus, the two resulting BAM files were merged using SAMtools v1.20 (Li et al., 2009). RNA‐editing sites were then identified and filtered using REDItools v2.0 (Picardi & Pesole, 2013), applying the following criteria: QUAL >30, depth >100×, and *p* < 0.1. Moreover, the identified RNA‐editing sites were categorized according to their function within the mitogenome and plastome, as detailed in Tables S2 and S3, respectively. For analyzing codon preference, the software MEGA v11 (Tamura et al., 2021) was employed to calculate the relative synonymous codon usage (RSCU) for all pt‐ and mt‐PCGs and to evaluate the codon usage bias with default settings.

### 2.4 Identification of repeat elements

The SSRs were detected by using the Perl program MISA (Beier et al., 2017), with the minimum repetition numbers of mono‐, di‐, tri‐, tetra‐, penta‐, and hexanucleotides 10, 5, 4, 3, 3, and 3, respectively. Additionally, the dispersed repeats, including forward, reverse, complement, and palindromic repeat sequences were identified by REPuter (Kurtz et al., 2001). The Hamming distance parameter was set to three, with a minimum repeat size requirement of 30.

### 2.5 Estimation of nucleotide substitution rates

KaKs_Calculator v2.0 (Wang et al., 2010) was utilized to calculate the pairwise Ka/Ks ratios for 56 pt‐ and 21 mt‐PCGs by analyzing the multiple nucleotide alignments of each gene pair. Additionally, the significance of the differences in the rates of synonymous mutations between the pt‐ and mt‐PCGs of *S. palustris* was evaluated using a *t*‐test in R v3.6.3.

### 2.6 Analysis of horizontal gene transfer (HGT)

To identify DNA fragments with potential horizontal transfer between the mitochondrial and plastid genomes in *S. palustris*, we employed the BLASTN program (Camacho et al., 2009) to search for homologous sequences across the two organelle genomes with an *E*‐value of 1e‐5 and a percent identity threshold of 80%. Then, all HiFi reads were remapped to organelle genomes using Minimap2 v2.28 (Li, 2021) with recommended settings. The boundaries of each transferred fragment sequence were then manually checked using IGV software (Thorvaldsdóttir et al., 2013). Additionally, we counted the 500 bp regions before and after the ends of the transfer fragment boundary.

### 2.7 Phylogenetic inference

A total of 26 representative species were selected for the phylogenetic analysis, comprising 14 monocots, seven dicots, two magnoliids (*Magnolia figo* and *Cinnamomum chekiangense*), one Chloranthales (*Hedyosmum orientale*), and two basal angiosperms (*Nymphaea colorata* and *Schisandra sphenanthera*) (Table S1). 56 and 21 shared PCGs extracted from mitogenomes and plastomes were aligned using MAFFT v7.490 (Katoh & Standley, 2013) employing the L‐INS‐i strategy, respectively. Ambiguous alignments were filtered using trimAl v1.2 (Capella‐Gutiérrez et al., 2009) with default parameters. The conserved alignment of pt‐ and mt‐PCGs was concatenated and used to construct the phylogenetic tree based on the maximum likelihood method implemented in IQ‐Tree v2.1.4 (Minh et al., 2020) following 1000 bootstrap replicates performed for each dataset.

## 3 RESULTS

### 3.1 Characterization of *S. palustris* organelle genomes

**FIGURE 1:**
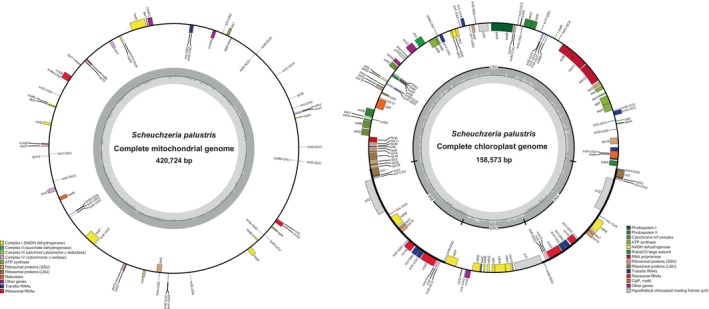
The organelle genomes map of *Scheuchzeria palustris*.

The mitogenome of *S. palustris* was assembled into a single circular molecule (Figure 1), spanning a total length of 420,724 bp, with a GC content of 47.33%. The annotation revealed 58 genes, comprising 30 protein‐coding genes (PCGs), 25 tRNA genes, and three rRNA genes (Table S2). Notably, six of the tRNA genes are present in duplicate: *trnA‐UGC*, *trnE‐UUC*, *trnfM‐CAU*, *trnI‐CAU*, *trnQ‐UUG*, and *trnY‐GUA*. The plastome measures 158,573 bp in length with a GC content of 37.31%. It is structured into inverted repeats of 25,311 bp, along with small single‐copy (SSC) and large single‐copy (LSC) regions measuring 20,070 bp and 87,881 bp, respectively (Figure 1). In total, 130 genes were annotated, including 85 PCGs, 37 tRNA genes, and eight rRNA genes (Table S3). Among these, six PCGs (*rpl2*, *rpl23*, *rps12*, *rps7*, *ndhB*, *ycf2*), four rRNA genes (*rrn16*, *rrn23*, *rrn4.5*, *rrn5*), and seven tRNA genes (*trnA‐UGC*, *trnI‐CAU*, *trnI‐GAU*, *trnL‐CAA*, *trnN‐GUU*, *trnR‐ACG*, *trnV‐GAC*) were identified as double‐copy genes.

### 3.2 Relative synonymous codon usage analysis

**FIGURE 2:**
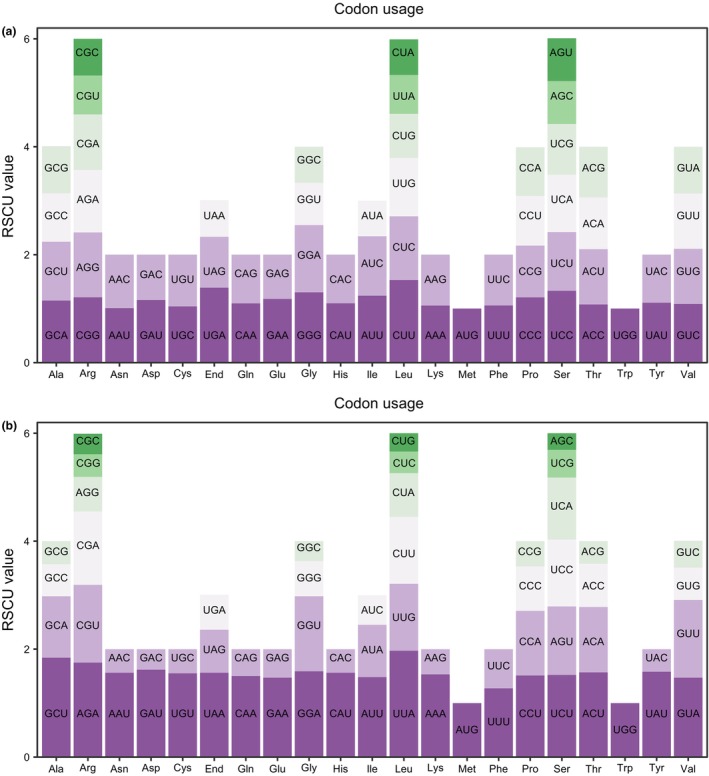
Analysis of RSCU of organelle genomes in *S. palustris*. (a) Analysis of RSCU in the *S. palustris* mitogenome. (b) Analysis of RSCU in the *S. palustris* plastome.

Codon usage frequencies for the 30 unique mt‐PCGs and 79 pt‐PCGs were estimated and RSCU values for each codon were presented in Figure 2. In the mitogenome of *S. palustris*, CUU (Leu) exhibited the highest RSCU value of 1.53, followed by UGA (End) at 1.39. In contrast, the codons with the lower RSCU value included CUA (Leu) and AUA (Ile) at 0.66. Unlike in the mitogenome, the plastome of *S. palustris*, UUA (Leu) showed the highest RSCU value for UUA (Leu) at 1.97, with the lowest recorded for AGC (Ser) at 0.31.

### 3.3 RNA editing and repeat elements analyses

**FIGURE 3:**
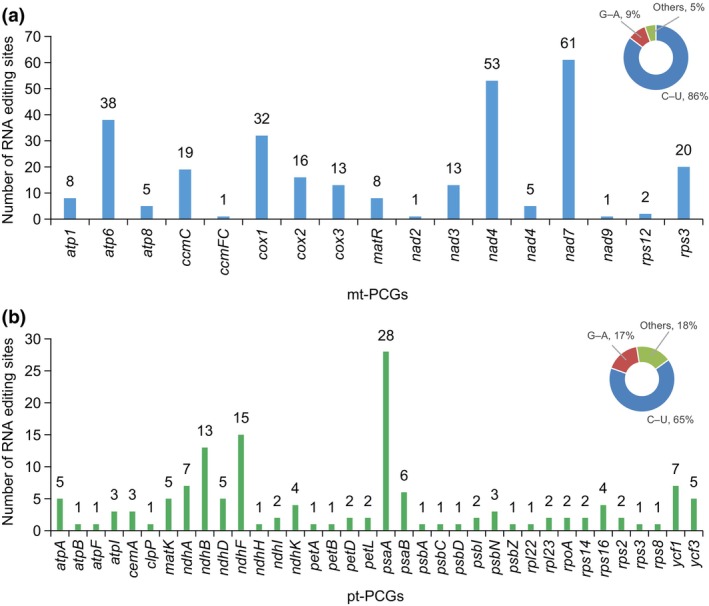
Characteristics of the RNA editing sites identified in PCGs of *S. palustris* organelle genome. (a) The distribution of RNA editing sites in mt‐PCGs. (b) The distribution of RNA editing sites in pt‐PCGs.

RNA editing is crucial for post‐transcriptional modifications within plant organelle genomes. Based on the transcriptome evidence, 296 RNA editing sites were identified across the 30 mt‐PCGs of *S. palustris* (Figure 3a). Among these, *nad7* had the highest number of RNA editing sites (61), while *ccmFC*, *nad2*, and *nad9* contained only one. Notably, 12 distinct types of RNA editing were identified in *S. palustris* mitogenome and predominantly characterized by C–U conversions, which accounted for 86% (254 sites) of the edits, while G–A took possession of 9% (27 sites). Similarly, in the plastome of *S. palustris*, 142 RNA editing sites were predicted, with *psaA* showing the highest frequency of RNA editing events (28 sites), and over half of these sites (65%) involved C–U conversions (Figure 3b).

**FIGURE 4:**
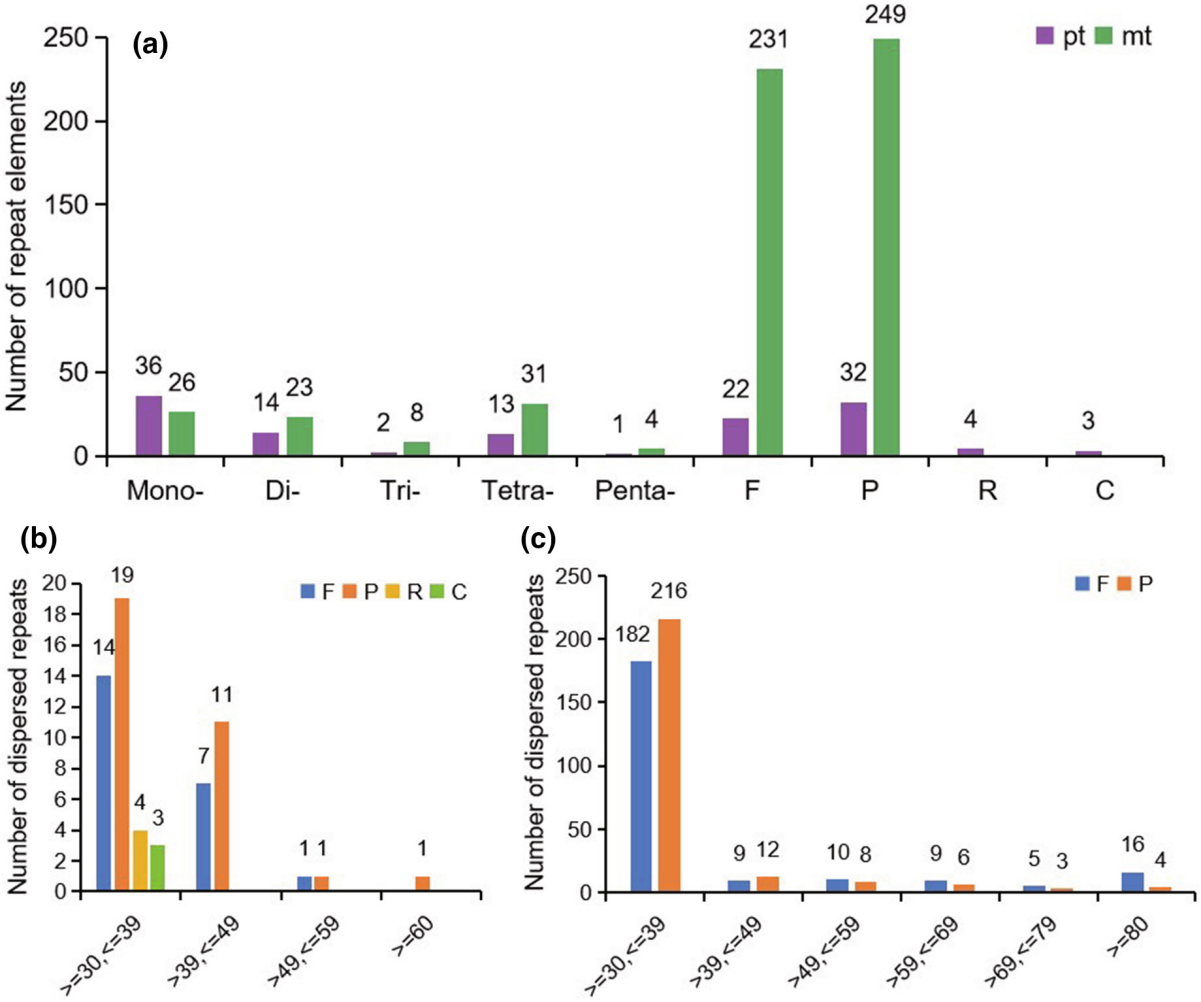
The distribution of SSR and dispersed repeats in the *S. palustris* organelle genomes. (a) The types of SSR and dispersed repeats; (b) and (c) represent the number of dispersed repeats in the plastome and mitogenome of *S. palustris*.

Analysis of repeat elements in the *S. palustris* mitogenome revealed a total of 92 simple sequence repeats (SSRs) and 480 dispersed repeats with lengths of at least 30 bp (Figure 4a; Figure 4c). In contrast, the plastome contained only 66 SSRs and 61 dispersed repeats. Among these, tetranucleotide SSRs were the most prevalent in the mitochondrial genome, constituting 31 (33.69%) of the total, whereas mononucleotide SSRs dominated in the chloroplast genome, representing 36 (54.54%) of the total. Regarding dispersed repeats, 231 were forward (F) and 249 were palindromic (P) in the mitochondrial genome. Notably, no reverse (R) or complementary (C) repeats were found, as shown in Figure 4a. The plastome contained 22 forward repeats, 32 palindromic repeats, four reverse repeats, and three complementary repeats, with the most common repeat lengths ranging from 30 to 39 bp (Figure 4b).

### 3.4 Homologous sequence analysis of two organelle genomes

**FIGURE 5:**
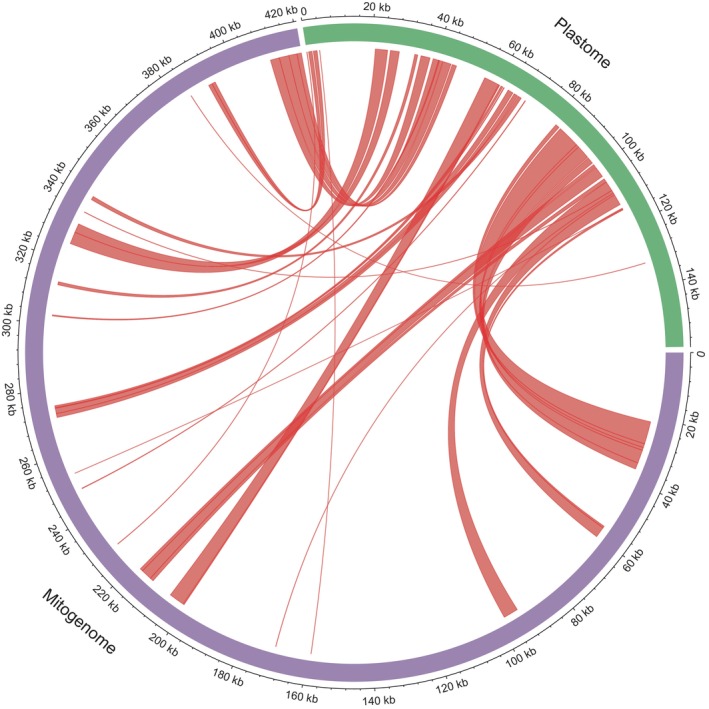
Homology analysis of the mitogenome and plastome of *S. palustris*.

A homology comparison between the mitogenome and plastome of *S. palustris* was illustrated in Figure 5. We identified 35 matching fragments that together accounted for 13.9% of the mitogenome, covering a total length of 58,479 bp with fragment sizes ranging from 32 to 6942 bp. Among these fragments, a total of 35 intact pt‐genes were functionally annotated, including 23 PCGs (*atpB*, *atpE*, *psaA*, *psbC*, *psbD*, *psbE*, *psbF*, *psbJ*, *psbL*, *rbcL*, *rpoC1*, *rps3*, *rps7*, *rps8*, *rps11*, *rps12*, *rps14*, *rps19*, *rpl2*, *rpl14*, *rpl16*, *rpl22*, *rpl23*), 12 tRNA genes (*trnA‐UGC*, *trnE‐UUC*, *trnfM‐CAU*, *trnH‐GUG*, *trnG‐GCC*, *trnI‐CAU*, *trnI‐GAU*, *trnN‐GUU*, *trnR‐ACG*, *trnV‐GAC*, *trnW‐CCA*, *trnY‐GUA*) and two rRNA genes (*rrn4.5* and *rrn5*).

### 3.5 The substitution rates of mt‐ and pt‐PCGs

**FIGURE 6:**
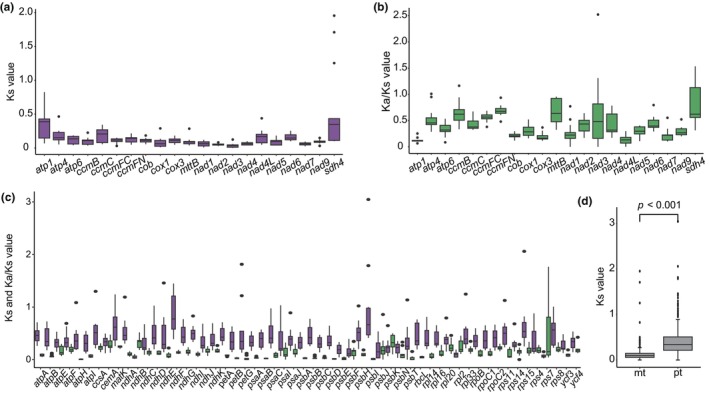
Boxplots of pairwise nucleotide substitution rates of *S. palustris* and other thirteen monocots. (a) Ks ratios in mt‐PCGs; (b) Ka/Ks ratios in mt‐PCGs; (c) Ks and Ka/Ks ratios in pt‐PCGs with purple representing Ks and green representing Ka/Ks; (d) Ks ratios between the two organelles.

We assessed the variation in synonymous mutation rates (*Ks*) within the mitogenome and plastome of *S. palustris* (Figure 6a,c). Our findings revealed a significant difference in the *Ks* ratios between the two organelles, with average values of 0.136 for mt‐PCGs and 0.392 for pt‐PCGs (Figure 6d). Moreover, comparing the pairwise *Ka*/*Ks* ratios among shared mt‐ and pt‐PCGs of *S. palustris* with those of 13 other monocots, we observed that most of the PCGs exhibited *Ka*/*Ks* ratios below 1, suggesting strong purifying selection during the evolution history (Figure 6b,c). Notably, the genes, including *atp4*, *ccmB*, *nad3*, and *sdh4* from the mitogenome, along with *rps7* from the plastome, displayed *Ka*/*Ks* ratios exceeding 1, implying that they might undergo positive selection.

### 3.6 Phylogenetic analysis by mitogenome and plastome

**FIGURE 7:**
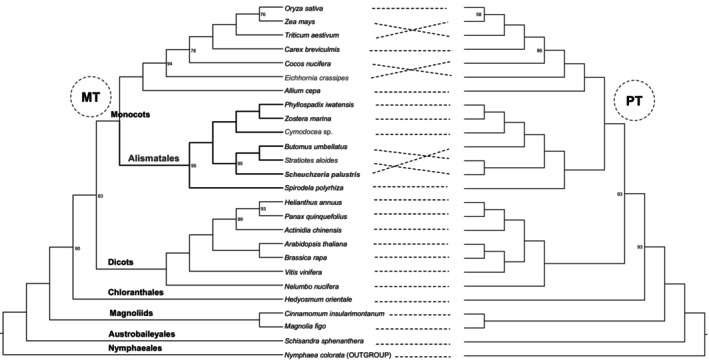
The phylogenetic relationships of *S. palustris* and 25 other species based on shared mt‐ and pt‐PCGs. Only the bootstrap value less than 100 was shown in the node.

To investigate the phylogenetic position of *S. palustris*, maximum likelihood trees were reconstructed for 26 species representing the main lineages of angiosperms using 21 mt‐PCGs and 56 pt‐PCGs separately. The resulting phylogenies for all selected species were well‐supported, with most nodes displaying robust bootstrap supports (BS = 100; Figure 7). However, the topologies of the plastid and mitochondrial trees exhibited slight differences, particularly within the Alismatales. The mitochondrial tree highly supported that *S. palustris* was sistered to *Butomus umbellatus* and *Stratiotes aloides* (BS = 95; Figure 7). In contrast, the plastid analysis indicated that *S. palustris* was a closer relationship between *S. palustris* and (*Phyllospadix* + *Zostera* + *Cymodocea*) (BS = 100; Figure 7).

## 4 DISCUSSION

Organelles in plants act as powerhouses for energy metabolism, supplying the essential capacity needed to sustain the life processes of plants (Maliga, 2022; Oldenburg & Bendich, 2015). Analyzing plant organelle genomes not only enhances our understanding of their functions, inheritance, and replication but also provides insights into their evolution and adaptation (Gutiérrez et al., 2023; Wang, Kan, et al., 2024; Wang, Liu, et al., 2024; Yang, Fu, et al., 2022; Yang, Zhao, et al., 2022). In our study, we successfully assembled the complete mitogenome and plastome of the threatened aquatic plant *S. palustris*. Consistent with the typical organelle genome structure in Alismatales (Chen et al., 2022; Petersen et al., 2017), both genomes are circular, with 420,724 bp and 158,573 bp in size, respectively. The notable size discrepancy between these organelle genomes may be attributed to an abundance of repetitive sequences, specifically dispersed repeats (mt: 480 vs. pt: 61; Table S5), in the mitogenome (Figure 4), which have been observed in other species, i.e., *Dystaenia takeshimana* (Park & Park, 2023). Although plant mitogenomes are typically large, they generally encode around 50–60 genes (Wang, Kan, et al., 2024; Wang, Liu, et al., 2024). The mitogenome of *S. palustris*, which is second only to *B. umbellatus* (450,826 bp) in size among the reported Alismatales, contains 52 unique genes (30 PCGs), which is close to that of other species with smaller size, such as *S. aloides* and *P. iwatensis*. Moreover, in angiosperms, GC content significantly influences amino acid composition and the structural maintenance of genomes during evolution (Wang, Kan, et al., 2024; Wang, Liu, et al., 2024; Zhou et al., 2014). Here, the GC content of the *S. palustris* mitogenome was approximately 47.33%, which is comparable to that of closely related species, with about 49.10% in *B. umbellatus* and 47.21% in *C. nodosa*. However, it was higher than the GC content in the plastome of *S. palustris* (ca. 37.31%), which may account for the observed differences in codon usage bias between PCGs of two organelle genomes (Figure 2).

RNA editing is a crucial post‐transcriptional modification in spermophytes that alters the genetic information in mRNAs, which can influence multiple biological functions, including the regulation of gene expression and adaptation to environmental changes (Edera et al., 2018; Szandar et al., 2022). Recently, increasing evidence has indicated that RNA editing is widely distributed among organelle genes, likely playing a key role in regulating genetics and energy metabolism (Li et al., 2019; Yang, Fu, et al., 2022; Yang, Zhao, et al., 2022). In our study, based on RNA‐seq data, we identified 296 RNA editing sites across 17 mitochondrial protein‐coding genes (mt‐PCGs), with *nad7* exhibiting the highest frequency at 61 sites, consistent with the typical range of RNA editing sites in angiosperms, approximately 300–500 (Edera et al., 2018). As a key component of the mitochondrial respiratory chain complex I, *nad7* primarily facilitates electron transfer and energy generation. The regulation of its RNA editing may significantly influence the efficiency of respiratory metabolism (Ramadan et al., 2022). In *S. palustris*, the RNA editing of *nad7* may contribute to its ability to survive in extreme environments and improve its adaptability to environments such as low temperature or high altitude. This editing could impact the overall growth and development of the plant by modifying the structure of the protein, enabling it to participate effectively in metabolic processes even under unfavorable conditions. Compared to mt‐PCGs, fewer RNA editing sites (142 sites) were observed in the pt‐PCGs of *S. palustris*, which is higher than those reported in most angiosperm plastomes (Zhang et al., 2023). Similar to previous studies (Chu & Wei, 2019; He et al., 2024), C‐to‐U was the predominant type of editing observed in both mt‐ (86%) and pt‐PCGs (65%) of *S. palustris*, potentially linked to adaptation to environmental conditions, such as cold temperatures and high altitudes. Moreover, the relatively high frequency of RNA editing in pt‐PCGs suggested that the regulation of photosynthesis‐related genes may play a more critical role in the adaptation and survival of *S. palustris* compared to genes involved in mitochondrial respiration. Additionally, genes undergoing positive selection are generally regarded as adaptations of plants to environmental stress during the evolution of plant organelle genomes (Eshel et al., 2022). In comparison to 13 other monocots, most mt‐ and pt‐PCGs undergo purifying selection (*Ka*/*Ks* < 1), however, four mt‐PCGs (*atp4*, *ccmB*, *nad3*, and *sdh4*) and one pt‐PCG (*rps7*) exhibit a high Ka/Ks ratio (>1), indicative of positive selection, which may be attributed to their adaptation to specific environmental conditions. Interestingly, *sdh4* plays a significant role in cellular responses to oxidative stress (Huang & Millar, 2013) and *rps7* encodes a ribosomal protein that plays a crucial role in protein synthesis within the cell (Chen et al., 2022), indicating these may assist *S. palustris* in adapting to specific environments.

Frequent rearrangements and horizontal gene transfers within organellar genomes have been widely proven to occur and play a pivotal role in plant evolution, which can result in the loss or gain of genes and changes in genome size (Cheng et al., 2021; Xu et al., 2023). In our study, a total of 35 mitochondrial plastid DNA (MTPT) with a total length of 58,479 bp were identified in *S. palustris*, accounting for 13.9% of mitogenome, which is significantly higher than most other monocots, such as *Apostasia shenzhenica* (5.12%; Ke et al., 2023) and *Eichhornia crassipes* (6.74%; He et al., 2024). The abundant repeat sequences in the mitogenome of *S. palustris* and may explain the high proportion of MTPT observed. Here, dispersed repeats were detected near most of the MTPT (Table S4), likely facilitating frequent interorganellar recombination, which can lead to variations in genome size and gene content (Cheng et al., 2021; Li et al., 2023). In line with previous studies (He et al., 2024; Xu et al., 2023), we identified 12 intact tRNA genes in MTPT (Table 1), supporting the frequent transfer of tRNA genes from chloroplasts to mitochondria. Interestingly, we found many intact pt‐PCGs (23) in MTPT, which may explain why pt‐PCGs of *S. palustris* have significantly more RNA editing sites compared to most flowering plants. Horizontal gene transfer (HGT) is a mechanism of genetic material transmission that has been identified as a significant driving force in biological evolution (Wang, Kan, et al., 2024; Wang, Liu, et al., 2024). The mitogenome of *S. palustris* contains several photosynthesis‐related genes derived from the plastome, which likely enhance the photosynthetic efficiency of *S. palustris* and facilitate its adaptation to lower‐temperature environments. Among these, *psaA*, which encodes for a protein subunit of photosystem I (PSI), has been proven to contribute to the adaptation of novel habitats and low temperatures (Gao et al., 2019; Song et al., 2020; Yu et al., 2023). Here, the highest RNA‐editing events (28 sites) were identified in *psaA* in intact PCGs of MTPT, which might contribute to the adaptation of *S. palustris* to unique habitats, such as low‐temperature and high‐altitude environments.

**TABLE 1:** Homologous fragments between mitogenome and plastome in Scheuchzeria palustris.

Fragments	Alightment length (bp)	Mitogenome			Plastome		
		Start	End	Contained genes	Start	End	Contained genes
mtpt1	6946	21,307	28,248	*trnI‐CAU, rpl16*	90,467	83,541	rps8, rpl14, rpl16, rps3, rpl22, rps19, rpl2, rpl23, trnI‐CAU, ycf2*
mtpt2	4707	201,137	205,822	\	55,656	60,340	atpE, atpB, rbcL, trnM‐CAU*
mtpt3	4561	93,265	97,815	*trnA‐UGC, trnI‐GAU*	109,604	105,052	trnI‐GAU, trnA‐UGC, rrn16*, rrn23*
mtpt4	4154	30,300	34,403	\	91,414	95,566	ycf2*
mtpt5	3740	325,267	329,006	\	20,978	24,717	rpoC1, rpoC2*
mtpt6	3300	213,764	217,063	\	102,921	99,625	rps7, rps12, ndhB*
mtpt7	3340	56,878	60,212	*trnN‐GUU, trnR‐ACG*	113,064	109,740	rrn4.5, rrn5, trnR‐ACG, trnN‐GUU, rrn23*
mtpt8	2993	413,799	416,785	*trnG‐GCC, trnfM‐CAU*	38,887	41,879	trnG‐GCC, trnfM‐CAU, rps14, psaB*
mtpt9	2798	411,009	413,806	\	35,086	37,883	*psbD, psbC*
mtpt10	2724	329,006	331,729	\	25,597	28,320	rpoC1*, ropB*
mtpt11	2541	416,773	419,305	\	42,002	44,540	psaA, psaB*
mtpt12	1984	34,404	36,387	\	95,862	97,845	ycf2*
mtpt13	1666	272,539	274,204	\	65,390	63,725	ycf4*, cemA*
mtpt14	1342	419,306	420,645	\	44,955	46,296	ycf3*
mtpt15	1201	28,239	29,439	\	82,958	81,758	rps11, rpoA*
mtpt16	1200	271,340	272,539	\	67,061	65,867	petA*
mtpt17	1133	390,625	391,756	\	3148	2016	trnK‐UUU*, matK*
mtpt18	1057	391,759	392,815	\	1608	552	psbA*
mtpt19	1100	212,665	213,763	*trnV‐GAC*	104,959	103,870	trnV‐GAC, rrn16*
mtpt20	906	217,046	217,951	\	99,384	98,479	ndhB*
mtpt21	906	312,129	313,034	*trnE‐UUC, trnY‐GUA*	34,026	33,121	*trnY‐GUA, trnE‐UUC*
mtpt22	878	29,432	30,309	\	90,496	91,367	ycf2*
mtpt23	591	274,372	274,962	\	61,808	61,218	accD*
mtpt24	1084	340,131	341,201	\	67,341	68,369	*psbJ, psbL, psbF, psbE*
mtpt25	547	56,338	56,884	\	114,918	114,372	ndhF*
mtpt26	245	200,735	200,979	\	60,714	60,470	accD*
mtpt27	168	274,204	274,371	\	62,327	62,160	\
mtpt28	335	302,483	302,776	\	40,866	40,533	psaB*
mtpt29	138	248,307	248,444	*trnW‐CCA*	69,791	69,927	*trnW‐CCA*
mtpt30	81	228,171	228,250	trnH‐GUG*	1	81	*trnH‐GUG*
mtpt31	91	335,726	335,809	\	108,128	108,038	trnA‐UGC*
mtpt32	66	169,820	169,885	rrn26*	108,987	109,052	rrn23*
mtpt33	49	384,390	384,438	\	132,471	132,519	ycf1*
mtpt34	45	158,852	158,896	\	3928	3972	trnK‐UUU*
mtpt35	32	253,456	253,487	\	114,682	114,651	ndhF*
Total	58,479						

*Note*: Asterisk labeled the genes that was incomplete.

Due to their maternally inherited, haploid nature, and ease of assembly, plant plastomes have been widely utilized to resolve evolutionary relationships across different lineages (Li et al., 2024; Mwanzia et al., 2020). In contrast, the challenges associated with obtaining plant mitogenomes have limited their use in phylogenetic studies (Guo et al., 2023; He et al., 2024). In this study, the phylogenetic relationships of the main lineages, as derived from both sets of organelle genomes, were consistent with the APG IV system and previous research (Hu et al., 2023; Li et al., 2019). Notably, in Alismatales, the placement of *S. palustris* showed conflict between the mitochondrial and plastid trees, which is likely due to significant differences in the substitution rates of mt‐ and pt‐PCGs in *S. palustris* (Figure 6; *p* < .001). Additionally, mt‐PCGs, generally associated with energy production, may be under stronger selective pressures compared to pt‐PCGs, potentially influencing their evolutionary rates, as supported by analyses of positive selection. However, due to the limited sampling of Alismatales (only seven species) in our study, we refrain from drawing firm conclusions until more mitogenomes from Alismatales become available. In addition, future work can include a comparative analysis of positively selected or RNA‐editing genes across closely related species to explore whether similar genomic patterns are observed in other regions, which can provide deeper insights into the adaptation strategies of aquatic species.

## 5 CONCLUSION

In this study, we assembled the complete organelle genomes of *Scheuchzeria palustris*, with sizes of 420,724 bp for the plastome and 158,573 bp for the mitogenome. Our results revealed a high abundance of RNA editing in pt‐PCGs, which may be attributed to frequent horizontal gene transfer within organellar genomes, playing a key role in rapid habitat adaptation. Five PCGs from the organellar genomes were detected under positive selection, potentially contributing to its unique adaptations to low‐temperature and high‐altitude environments. Additionally, phylogenomic analyses based on pt‐ and mt‐PCGs indicated conflicting positions of *S. palustris* within Alismatales, suggesting significant differences in the substitution rates of mt‐ and pt‐PCGs in this species. The organelle genomes of the threatened aquatic plant *S. palustris* provide vital genomic resources and scientific support for its future conservation.

## AUTHOR CONTRIBUTIONS

**Zhi‐Zhong Li:** Conceptualization (lead); formal analysis (equal); supervision (equal). **Jin‐Ming Chen:** Supervision (equal). **Xiang‐Yan He:** Formal analysis (equal); investigation (equal); writing – original draft (equal).

## CONFLICT OF INTEREST STATEMENT

The authors declare that they have no conflict of interest.

## Supporting information


Table S1.

Table S2.

Table S3.

Table S4.

Table S5.

## Data Availability

The mitogenome and plastome of *Scheuchzeria palustris* have been deposited in NCBI with accession Nos. PQ031343 and PQ031344. The raw sequencing data are available at https://doi.org/10.6084/m9.figshare.26176834.v1.
