# Supplementary material for: Complete organelle genomes of the threatened aquatic species Scheuchzeria palustris (Scheuchzeriaceae): Insights into adaptation and phylogenomic placement
Source: Ecol Evol. 2024 Aug 31;14(9):e70248. doi: 10.1002/ece3.70248 (PMC11364858; doi:10.1002/ece3.70248)
Supplement: Supplementary file 1 — Table S1. [file ECE3-14-e70248-s003.docx]

**Table S1** Information on plastomes and mitogenomes was retrieved from public databases and used for phylogenetic analyses in this study.

| Group | Species | Mitogenome GenBank Accession No. | Plastome GenBank Accession No. |
| --- | --- | --- | --- |
| Angiosperm basal group | *Nymphaea colorata* | NC_037468.1 | NC_057562.1 |
|  | *Schisandra sphenanthera* | NC_042758.1 | NC_037145.1 |
| Magnoliids | *Magnolia figo* | NC_082234.1 | NC_053861.1 |
|  | *Cinnamomum chekiangense* | NC_082065.1 | OR360835.1 |
| Chloranthales | *Hedyosmum orientale* | NC_082064.1 | Li et al., 2021 |
| Monocots | *Spirodela polyrhiza* | NC_017840.1 | NC_015891.1 |
|  | *Butomus umbellatus* | NC_021399.1 | NC_051949.1 |
|  | *Stratiotes aloides* | NC_035317.1 | Li et al., 2022 |
|  | *Zostera marina* | NC_035345.1 | NC_036014.1 |
|  | *Phyllospadix iwatensis* | NC_068804.1 | NC_058622.1 |
|  | *Allium cepa* | NC_030100.1 | NC_024813.1 |
|  | *Cocos nucifera* | NC_031696.1 | NC_022417.1 |
|  | *Oryza sativa* | NC_007886.1 | NC_031333.1 |
|  | *Zea mays* | NC_007982.1 | NC_001666.2 |
|  | *Triticum aestivum* | NC_036024.1 | NC_002762.1 |
|  | *Carex breviculmis* | NC_068626.1 | NC_072263.1 |
|  | Eichhornia crassipes | PP112345 | NC_046773.1 |
|  | *Cymodocea* sp. | Ma et al., 2024 | Li et al., 2023 |
|  | Scheuchzeria palustris | PQ031343 | PQ031344 |
| Dicots | *Nelumbo nucifera* | NC_030753.1 | NC_025339.1 |
|  | *Vitis vinifera* | NC_012119.1 | NC_007957.1 |
|  | *Arabidopsis thaliana* | NC_037304.1 | NC_000932.1 |
| Dicots | *Brassica rapa* | NC_049892.1 | NC_040849.1 |
|  | *Actinidia chinensis* | NC_065997.1 | NC_026690.1 |
|  | *Helianthus annuus* | NC_023337.1 | NC_007977.1 |
|  | *Panax quinquefolius* | NC_067574.1 | NC_027456.1 |

**References:**

Li HT, Luo Y, Gan L et al (2021). Plastid phylogenomic insights into relationships of all flowering plant families. BMC Biol 19(1): 1-13.

Li ZZ, Lehtonen S, Martins K et al (2022). Complete genus-level plastid phylogenomics of Alismataceae with revisited historical biogeography. Mol Phylogenet Evol 166: 107334.

Li ZZ, Lehtonen S, Chen JM (2023). The dynamic history of plastome structure across aquatic subclass Alismatidae. BMC Plant Biol 23:125.

Ma X, Vanneste S, Chang J et al (2024) Seagrass genomes reveal ancient polyploidy and adaptations to the marine environment. Nat Plants 10(2):240-255.
