# Supplementary material for: Complete organelle genomes of the threatened aquatic species Scheuchzeria palustris (Scheuchzeriaceae): Insights into adaptation and phylogenomic placement
Source: Ecol Evol. 2024 Aug 31;14(9):e70248. doi: 10.1002/ece3.70248 (PMC11364858; doi:10.1002/ece3.70248)
Supplement: Supplementary file 2 — Table S2. [file ECE3-14-e70248-s001.docx]

**Table S2** Gene profile of the *Scheuchzeria palustris* mitogenome

| Group of genes | Name of genes |
| --- | --- |
| ATPase | *atp1, atp4, atp6, atp8* |
| Cytochrome c biogenesis | *ccmB, ccmC, ccmFC, ccmFN* |
| Apocytochrome b | *cob* |
| cytochrome c oxidase | *cox1, cox2, cox3* |
| Maturase R | *matR* |
| Transport membrane protein | *mttB* |
| NADH dehydrogenase | *nad1, nad2, nad3, nad4, nad4L, nad5, nad6, nad7, nad9* |
| Small subunit of ribosome | *rps1, rps11, rps12, rps3* |
| Large subunit of ribosome | *rpl10, rpl16* |
| succinate dehydrogenase | *sdh4* |
| rRNA | *rrn18, rrn26, rrn5* |
| tRNA | *trnA-UGC(×2), trnC-GCA, trnD-GUC, trnE-UUC(×2), trnfM-CAU(×2), trnG-GCC, trnH-GUG, trnI-CAU(×2), trnI-GAU, trnK-UUU, trnN-GUU, trnP-UGG, trnQ-UUG(×2), trnR-ACG, trnR-CCU, trnS-UGA, trnV-GAC, trnW-CCA, trnY-GUA(×2)* |
