# Supplementary material for: Complete organelle genomes of the threatened aquatic species Scheuchzeria palustris (Scheuchzeriaceae): Insights into adaptation and phylogenomic placement
Source: Ecol Evol. 2024 Aug 31;14(9):e70248. doi: 10.1002/ece3.70248 (PMC11364858; doi:10.1002/ece3.70248)
Supplement: Supplementary file 3 — Table S3. [file ECE3-14-e70248-s005.docx]

**Table S3** Gene profile of the *Scheuchzeria palustris* plastome

| Group of functional genes | Name of genes |
| --- | --- |
| Photosystem l | *psaA, psaB, psaC, psaI, psaJ* |
| Photosystem ll | *psbA, psbB, psbC, psbD, psbE, psbF, psbH, psbI, psbJ, psbK, psbL, psbM, psbN, psbT, psbZ* |
| Cytochrome B f complex | *petA, petB, petD, petG, petL, petN* |
| ATP synthase | *atpA, atpB, atpE, atpF, atpH, atpI* |
| Rubisco large subunit | *rbcL* |
| RNA polymerase | *rpoA, rpoB, rpoC1, rpoC2* |
| Ribosomal proteins large subunit | *rpl14, rpl16, rpl2(×2), rpl20, rpl22, rpl23(×2), rpl32, rpl33, rpl36* |
| Ribosomal proteins small subunit | *rps11, rps12(×2), rps14, rps15, rps16, rps18, rps19, rps2, rps3, rps4, rps7(×2), rps8* |
| NADH dehydrogenase | *ndhA, ndhB(×2), ndhC, ndhD, ndhE, ndhF, ndhG, ndhH, ndhI, ndhJ, ndhK* |
| Other genes (OG) |  |
| Conserved coding frame | *ycf1, ycf2(×2), ycf3, ycf4* |
| Acetyl-CoA-carboxylase | *accD* |
| ATP-dependent protease | *clpP* |
| Translation initiation factor | *infA* |
| Cytochrome c biogenesis | *ccsA* |
| Membrane protein | *cemA* |
| Maturase | *matK* |
| rRNA | *rrn16(×2), rrn23(×2), rrn4.5(×2), rrn5(×2)* |
| tRNA | *trnA-UGC(×2), trnC-GCA, trnD-GUC, trnE-UUC, trnF-GAA, trnG-GCC, trnG-UCC, trnH-GUG, trnI-CAU(×2), trnI-GAU(×2), trnK-UUU, trnL-CAA(×2), trnL-UAA, trnL-UAG, trnM-CAU, trnN-GUU(×2), trnP-UGG, trnQ-UUG, trnR-ACG(×2), trnR-UCU, trnS-GCU, trnS-GGA, trnS-UGA, trnT-GGU, trnT-UGU, trnV-GAC(×2), trnV-UAC, trnW-CCA, trnY-GUA, trnfM-CAU* |
