# Supplementary material for: Complete organelle genomes of the threatened aquatic species Scheuchzeria palustris (Scheuchzeriaceae): Insights into adaptation and phylogenomic placement
Source: Ecol Evol. 2024 Aug 31;14(9):e70248. doi: 10.1002/ece3.70248 (PMC11364858; doi:10.1002/ece3.70248)
Supplement: Supplementary file 4 — Table S4. [file ECE3-14-e70248-s004.docx]

**Table S4** Repetitive sequences near the homologous fragments ± 500 bp between mitogenome and plastome in *Scheuchzeria palustris*

| Fragments | Mt_strat ± 500bp | Mt_stop ± 500bp | Pt_strat ± 500bp | Pt_stop ± 500bp |
| --- | --- | --- | --- | --- |
|  | Position length type | Position length type | Position length type | Position length type |
| mtpt1 | 21696, 46, F | \ | \ | 90016, 31, P 90031, 46, P 90046, 31, P |
| mtpt2 | \ | 205571, 30, F | \ | 60193, 30, C 60196, 33, R 60197, 30, P 60198, 30, F 60199, 31, R 60200, 31, C |
| mtpt3 | 93532, 30, P | 97317, 74, P 97751, 33, P | \ | \ |
| mtpt4 | \ | \ | \ | 95118, 33, P 95142, 33, P |
| mtpt5 | \ | \ | \ | \ |
| mtpt6 | 214127, 37, P | \ | \ | \ |
| mtpt7 | 56573, 32, F | 60210, 38, F | \ | \ |
| mtpt8 | \ | \ | 38787, 30, R | \ |
| mtpt9 | \ | \ | \ | 37977, 32, P |
| mtpt10 | \ | 331725, 31, F 331753, 36, F 331757, 35, F 331758, 31, F | \ | \ |
| mtpt11 | \ | \ | \ | \ |
| mtpt12 | \ | \ | \ | \ |
| mtpt13 | \ | \ | \ | \ |
| mtpt14 | \ | \ | \ | 46222, 37, F 46234, 30, F |
| mtpt15 | \ | \ | \ | \ |
| mtpt16 | \ | \ | \ | 67251, 31, P |
| mtpt17 | \ | \ | 1751, 46, P | \ |
| mtpt18 | \ | \ | \ | 46, 1751, P |
| mtpt19 | 212679, 33, P | 214127, 37, P | \ | \ |
| mtpt20 | \ | \ | 98466, 49, P | \ |
| mtpt21 | \ | 313322, 30, F | \ | \ |
| mtpt22 | \ | \ | 90016, 31, P 90031, 46, P 90046, 31, P | \ |
| mtpt23 | \ | \ | \ | \ |
| mtpt24 | \ | 341388, 30, P | 67251, 31, P | \ |
| mtpt25 | 56573, 32, F | \ | \ | \ |
| mtpt26 | 200346, 32, P | \ | 60193, 30, C 60196, 33, R 60197, 30, P 60198, 30, F 60199, 31, R 60200, 31, C | \ |
| mtpt27 | \ | \ | \ | \ |
| mtpt28 | 302482, 30, P | 302519, 30, P 302640, 40, P 302653, 65 P 302683, 60 P 302719, 31 P | \ | \ |
| mtpt29 | \ | 248721, 31, F | \ | \ |
| mtpt30 | \ | \ | \ | \ |
| mtpt31 | 335762, 47, F 335775, 34, F | \ | \ | \ |
| mtpt32 | 169388, 31, P 169819, 59, P 170143, 30, P | \ | \ | \ |
| mtpt33 | 384294, 50, F | \ | \ | \ |
| mtpt34 | 158610, 241, P | \ | \ | \ |
| mtpt35 | 253309, 31, P 253311, 37, P 253327, 31, P 253364, 36, P 253411, 30, P 253455, 32, F | 253654, 33, F | \ | \ |
