# Supplementary material for: Complete organelle genomes of the threatened aquatic species Scheuchzeria palustris (Scheuchzeriaceae): Insights into adaptation and phylogenomic placement
Source: Ecol Evol. 2024 Aug 31;14(9):e70248. doi: 10.1002/ece3.70248 (PMC11364858; doi:10.1002/ece3.70248)
Supplement: Supplementary file 5 — Table S5. [file ECE3-14-e70248-s002.docx]

**Table S5** The key differences between the plastome and mitogenome in *Scheuchzeria palustris*

|  | Mitogenome | Plastome |
| --- | --- | --- |
| Total length (bp) | 420,724 | 158,573 |
| Total genes | 58 | 130 |
| PCGs | 30 | 85 |
| unique PCGs | 30 | 79 |
| tRNA genes | 25 | 37 |
| rRNA genes | 3 | 8 |
| RNA editing sites | 296 | 142 |
| Simple sequence repeats | 92 | 66 |
| Dispersed repeats | 480 | 61 |
